# Is CCNU (lomustine) valuable for treatment of cutaneous epitheliotropic lymphoma in dogs? A critically appraised topic

**DOI:** 10.1186/s12917-017-0978-7

**Published:** 2017-02-21

**Authors:** Aurore Laprais, Thierry Olivry

**Affiliations:** 1 Department of Clinical Sciences, College of Veterinary Medicine, North Carolina State University, Raleigh, NC USA; 2 Comparative Medicine Institute, College of Veterinary Medicine, North Carolina State University, Raleigh, NC USA

**Keywords:** Canine, Chemotherapy, Dog, Epitheliotropic, Lomustine, Lymphosarcoma, Mycosis fungoides, Neoplasia

## Abstract

**Background:**

CCNU and other treatment protocols are commonly offered to owners for the treatment of dogs diagnosed with cutaneous (epitheliotropic) T-cell lymphoma (CTCL). Chemotherapy protocols provide variable benefits; they have different side-effects, and they typically require monitoring to detect drug toxicity at a non-negligible cost to the owner. At this time, even though CCNU is most often recommended to treat dogs with CTCL, there is no clear consensus on the benefit of this drug. Knowing which chemotherapy protocol yields the highest rate of complete remission and longest survival times would help veterinarians and pet owners select treatment options based on the best evidence available. Our objective was to review the literature to compare the complete remission rates and survival times of CCNU-based protocols to those of other interventions. We critically assessed the data included in articles reporting treatment outcome in at least five dogs with CTCL. Single case reports and case series with less than five patients were not reviewed to avoid anecdotal evidence of lower quality.

**Results:**

The search for, and review and analysis of, the best evidence available as of February 8, 2017, suggests that CCNU and pegylated liposomal doxorubicin appear to yield the highest rate of complete remission in approximately one-third of dogs with CTCL. Other treatment protocols did not report usable information on remission rates. Without any treatment, the mean/median survival time in dogs with CTCL varied between 3 and 5 months. With CCNU protocols, the median survival time was 6 months and the one with retinoids (isotretinoin and/or etretinate), PEG L-asparaginase or prednisolone monotherapy was 11, 9 and 4 months, respectively; all these durations were obtained from small numbers of dogs, however.

**Conclusions:**

CCNU leads to a complete remission of signs in approximately one-third of dogs with CTCL, but such remissions are of short duration. The median survival time after CCNU appears longer than that without treatment, but other drugs appear to provide a better long-term prognosis. Further studies are required to investigate the effect of CCNU, alone or in combination, on remission rates, survival times and impact on quality of life.

## Background

Lomustine (CCNU) or other chemotherapy protocols are commonly offered to owners for the treatment of their dogs diagnosed with cutaneous (epitheliotropic) T-cell lymphoma (CTCL). Although these regimens are expected to lead to a temporary partial or complete remission (CR) of skin lesions, monitoring for the detection of common severe adverse events requires regular laboratory monitoring that might represent a significant financial burden to the clients [1]. At this time, there is no clear consensus on the benefit of the various treatment regimens for dogs with CTCL.

Knowing which chemotherapy protocol has the strongest evidence for yielding the best complete remission rates and longest survival times would help veterinarians and pet owners select treatment options based on the best evidence available. Our objective was to review the literature to compare the complete remission rates and survival times of CCNU-based protocols to those of other interventions.

### Clinical scenarios

You have two patients: The first one is a 5-year-old female Labrador retriever crossbred dog. The owners report that the lesions started 2.5 months beforehand and that they consisted, at first, of generalized scaling and pruritus. They evolved into multifocal areas of truncal alopecia and erythema along with an ulcer on the caudal abdomen. The dog was placed on a tapering course of prednisone that led to a reduction in clinical signs. After discontinuing the steroids, the dog developed alopecic erythematous macules, patches and plaques, and she became more pruritic than before.

Your second patient is a 12-year-old male golden retriever presenting with multifocal large patchy areas of hypopigmentation and depigmentation along with multifocal erythematous plaques and nodules. The owner reports that the dog may have had depigmented muzzle lesions for the past 2 years.

In both cases, skin biopsies confirmed your suspicion of CTCL. You wonder which chemotherapeutic option would be most beneficial for these two dogs.

### Structured question

*In a dog with CTCL, which chemotherapeutic treatment protocol would be most effective to induce the CR of clinical signs and to lead to the longest survival?*

## Methods

### Search strategy

The CAB Abstracts and Web of Science (WoS) Science Citation Index Expanded databases were searched on April 26, 2016 for relevant articles using the following string: (dog or dogs or canine) and (cutaneous or epitheliotropic or epidermotropic) and (lymphoma or lymphosarcoma) and (CCNU or lomustine or vincristine or cyclophosphamide or chlorambucil or predniso* or chemotherap*). The search was limited to the period 1980–2016. We then screened the bibliographies of identified articles for additional relevant reports. At the time of revision of this manuscript (Febuary 08, 2017), a second broad literature search was done to identify papers published since the first search.

## Results and discussion

### Identified evidence

Our literature search found 63 and 65 articles in the CAB Abstract and Web of Science (WoS) databases, respectively. Citations were initially examined for the identification of articles reporting the outcome of treatment in dogs with CTCL diagnosed via histopathology. We then evaluated abstracts, and articles with potentially relevant information were read in full. The bibliography of these papers was analyzed further for additional pertinent reports.

We only selected articles that provided definite information on the rate of CR /or survival times after diagnosis or treatment of at least five dogs with CTCL; articles and information on partial remission were not reviewed further.

Two retrospective [2, 3] and one prospective studies [4] providing clear information on CR rates in dogs with CTCL were found in both CAB and WoS databases. One preclinical trial [5] and one pilot study [6] were subsequently added following the review of the bibliographies of articles identified in the electronic searches. Finally, we added one last article that had been published after the performance of our initial literature search [7].

For survival times after diagnosis or treatment, one retrospective study [8] and one prospective study [9] were identified in the CAB abstracts, one of which was also found in the WoS [8]. A prospective [10] and a retrospective study [11] were added following the review of bibliographies of articles found in the electronic search.

### Evaluation of evidence

#### Complete remission rates

The rates of CR in studies involving five or more dogs were given for protocols using CCNU, pegylated liposomal doxorubicin, the prodrug VDC-1101 (a guanine analog) and masitinib (Table 1).

**Table 1:** Rates of complete remission in dogs with CTCL

	CCNU		Pegylated liposomal doxorubicin		VDC-1101 and prednisone		Masitinib	
References	N	CR (N, %)	N	CR (N, %)	N	CR (N, %)	N	CR (N, %)
Morges, 2014 [4]					10	0 (0%)		
Risbon, 2006 [2]	46^a^	15 (33%)						
Williams, 2006 [3]	36	6^b^ (33%)						
Graham, 1999 [6]	5	5^c^ (100%)						
Vail, 1997 [5]			9	3 (33%)				
Holtermann, 2016 [7]							10^d^	2 (20%)
**TOTAL**	87	26 (30%)	9	3 (33%)	10	0 (0%)	1-	2 (20%)

*Abbreviations*: *CR* complete remission, *N* number of dogs

^a^14 dogs received CCNU monotherapy, 27 also received glucocorticoids that were later either tapered, discontinued or maintained. The co-administration of prednisone was reported not to be significantly associated with the response or its duration. Other concurrent medications included PEG L-asparaginase (6 dogs), essential fatty acids (8), nonsteroidal anti-inflammatory drugs (3), retinoids (2), and interferon (1)

^b^67% of the dogs experiencing a CR also received concurrent glucocorticoids

^c^2 dogs may have received concurrent surgery

^d^the response was assessed on the three most dominant “target” lesions

Altogether, CCNU, alone or in conjunction with other concurrent medications, yielded a combined rate of CR of 30% of the 87 dogs reported [2, 3, 6]. The median duration of CR, estimated from only 15 dogs in one article [2], was 132 days (range: 26–258 days). The time to achieve CR was not identified in any article. In one report of 46 dogs [2], the overall median number of treatments needed to observe a response (type not specified) was one (range: one to six cycles).

A similar rate of CR occurred with pegylated liposomal doxorubicin, but the number of dogs treated (nine) was low [5]. The median duration of CR was shorter (90 days, range: 21–340) than with CCNU; the time to CR was not reported either.

The prednisone and VDC-1101 combination did not lead to CR in any of the ten dogs to which it was administered [4].

Finally, masitinib induced a CR of signs of CTCL in 2/10 treated dogs (20%); this remission lasted for 126 days in one dog and over 3 years in the other [7].

### Survival times after diagnosis or treatment initiation

The survival times were only available for a few dogs treated with each intervention (Table 2). Without any treatment, the median survival time in dogs with CTCL varied between 3 [11] and 5 months [8].

**Table 2:** Survival times in dogs with CTCL (in months)

	CCNU		Prednisolone		Retinoids^a^		PEG L-asparaginase^b^		No treatment	
References	N	S	N	S	N	S	N	S	N	S
Fontaine, 2010 [8]^c^	7	6	6	4					3	5
White, 1993 [9]^c^					5	11				
Moriello, 1993 [10]^d^							7	9		
Beale, 1993 [11]^e^									8	3

*Abbreviations*: *N* number, *S* median or mean survival times (in months) after diagnosis or treatment

^a^the concurrent use of glucocorticoids was allowed

^b^three patients also received glucocorticoids, one patient was also treated with vincristine/cyclophosphamide and doxorubicin

^c^Most dogs were euthanized; the specific number of dogs was not specified

^d^All dogs were euthanized

^e^the number of dogs euthanized was not specified

The median/mean survival times were, in order of decreasing durations, 11 months after treatment with the retinoids isotretinoin and/or etretinate [9], 9 months after PEG L-asparaginase [10], 6 months after CCNU protocols [8] and 4 months after prednisolone alone [8].

### Limitations

The main limitation of this compilation is that any confounding effect of the dog’s age, CTCL severity, CTCL stage or time before the diagnosis was made or treatment initiated were not evaluated for their possible impact on CR or survival times in any of these studies. Another limitation is that CCNU was used alone in only one-third of dogs in the largest case series [2, 3], and the rates of CR were not compared between dogs receiving CCNU monotherapy and those treated with combination regimens. Furthermore, the survival times provided were likely to have been influenced by euthanasia decisions by the owner, thereby not reflecting disease-induced death. Finally, many of the estimates provided herein were only assembled from a small number of patients.

## Conclusions

### Implications for practitioners

In spite of the limitations raised above, and taking into consideration the best evidence available, CCNU protocols, when used alone or in combination with other drugs, appear to lead to CR rates of 30%, but the median duration of such CRs remains unclear. The median survival time of CTCL after CCNU protocols is 6 months following diagnosis. Retinoids or PEG L-asparaginase appear to offer longer median survival times, but these drugs are not routinely available.

At the time of this writing, CCNU—alone or in combination with other drugs—appears to be a valuable option to treat dogs with CTCL; unfortunately, the common occurrence of myelopoietic and/or gastrointestinal side effects requires frequent blood test monitoring that increases the cost of treatment.

### Implication for research

Additional studies are needed to compare the rates of CR and survival times using glucocorticoids, CCNU or retinoids (e.g. bexarotene), either alone or in various combinations thereof. Because of the potential toxicity and side effects of CCNU, the quality of life in dogs receiving CCNU protocols should be compared to that without any treatment or with other medications. Future studies should also attempt to compare CR rates and survival times in dogs of various age groups as well as in those with variable duration of clinical signs, stages or severities of CTCL.

## Abbreviations

CCNU: 1-(2-chloroethyl)-3-cyclohexyl-1-nitrosourea (i.e. lomustine)
CR: complete remission
CTCL: cutaneous T-cell lymphoma
PEG: polyethylene glycol
